# Novel subtype of coxitis knee associated with acetabular dysplasia of the hip: a case series

**DOI:** 10.1186/s42836-023-00225-z

**Published:** 2024-02-03

**Authors:** Patricio III Dumlao, Kiminori Yukata, Yutaka Suetomi, Atsunori Tokushige, Takashi Sakai, Hiroshi Fujii

**Affiliations:** 1 Ogori Daiichi General Hospital, 862-3 Ogori Shimogo, Yamaguchi City, 754-0002 Japan; 2 Department of Orthopaedic Surgery, Yamaguchi University, 1-1-1, Minamikogushi, Ube City, Yamaguchi, 755-8505 Japan

**Keywords:** Acetabular dysplasia, Coxitis knee, Gonarthritis, Multiple joint arthritis

## Abstract

**Background:**

Multiple joint arthritis patterns require a comprehensive understanding to optimize patient management. This study aimed to present a patient cohort that deviated from known definitions of coxitis knee (CK), identifying and characterizing this atypical group.

**Methods:**

Patients undergoing both total hip arthroplasty and total knee arthroplasty between January 2008 and December 2018 were retrospectively reviewed. The patients were classified into a typical coxitis knee group (classic, long leg arthropathy, and windswept deformity) and an atypical coxitis knee group. Leg-length discrepancy, body mass index (BMI), and radiographic parameters of the groups were compared and analyzed.

**Results:**

A total of 31 patients were allocated to the typical coxitis knee group (*n* = 10), and atypical coxitis knee group (*n* = 21). In the atypical group, 27 hips were involved, of which 21 had acetabular dysplasia, 5 exhibited subchondral insufficiency fracture-like changes, and only 1 had classic osteoarthritis. Among the 27 knees undergoing total knee arthroplasty, 26 showed varus alignment, 1 was within the normal range, and none was valgus. Acetabular dysplasia involved ipsilateral (*n* = 1), contralateral (*n* = 14), and bilateral (*n* = 6) hips, showing atypical coxitis knee. Patients with acetabular dysplasia were more likely to exhibit atypical CK.

**Conclusion:**

Most patients in the cohort displayed acetabular dysplasia and contralateral varus knees, constituting a pattern referred to as acetabular dysplasia-associated gonarthritis. Identifying this novel subtype may have important clinical implications for regions with high risk factors, where acetabular dysplasia and constitutional genu varum are prevalent.

**Graphical Abstract:**

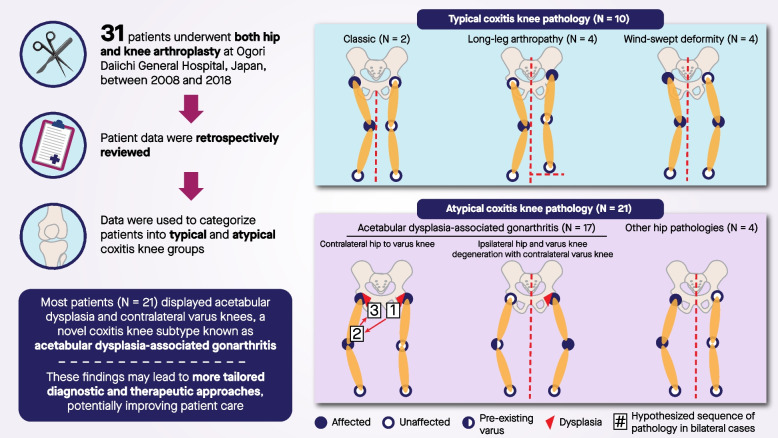

## Introduction

Osteoarthritis is a complex disease that may affect multiple joints, significantly impacting patients’ quality of life [1]. The multiple joint osteoarthritis affects about 5% to 25% of patients and is relatively under-studied as a distinct entity [2]. However, it is of great importance to address multiple joint osteoarthritis since it poses severe burden on quality of life, personal activity, and life expectancy as compared to monoarthritis [2].

There are several multiple joint osteoarthritis-related conditions, including hip-spine syndrome, knee-spine syndrome, and hip and knee osteoarthritis [3–5]. Among them, the prevalence and associated factors of hip and knee osteoarthritis are comparatively less understood [5–8]. Though the effect of a pathologic hip joint, or a hip with risk factors in the development of knee osteoarthritis has been defined as “Coxitis Knee” (CK) [9, 10]. The original criteria for classic CK, involving an ankylosed hip due to a pathological or surgical cause with no prior knee pathology, have expanded to include “Long Leg Arthropathy” (LLA) and “Wind-Swept Deformity” (WSD) variants, now included hip adduction contracture and leg length discrepancies exceeding 2.5 cm [9, 11–14]. Nonetheless, there is a set of patients with hip or knee pathologies that do not follow the classic pattern and cannot be explained by the above causes. In this context, acetabular dysplasia of the hip, a recognized contributor to osteoarthritis, has a global incidence varying from 1% to 10% [15]. It is notably more prevalent in the Asian population, affecting up to 70% of hip osteoarthritis patients in different regions [15, 16].

Our study aimed to investigate the association between hip acetabular dysplasia and knee osteoarthritis, distinct from typical coxitis knee. We focused on patients who underwent both total hip arthroplasty (THA) and total knee arthroplasty (TKA) to determine atypical CK incidence, compared it to typical CK variants, and assessed the distinctive clinical and radiological features in affected joints.

## Materials and methods

We retrospectively reviewed 1411 patients who underwent hip or knee arthroplasty at our hospital between 2008 and 2018. We identified 31 patients who underwent both THA and TKA. The mean age at surgery was 74.5 (56–86) years, with 29 female patients and 2 male patients. These patients were further categorized into distinct typical and atypical CK groups, including classic CK, LLA, and WSD. A breakdown of CK classification is presented in Table 1.

**Table 1:** Demographics of the coxitis knee for 31 patients

		**Coxitis knee classification**				
	Total (*n* = 31)	Typical (*n* = 10)			Atypical (*n* = 21)	Cohen’s d effect (Typical vs. Atypical)^a^
Characteristic		Classic *n* = 2)	LLA (*n* = 4)	WSD (*n* = 4)		
Age (year) (mean ± SD)	74.5 ± 7.9	60 ± 4	75.8 ± 9.3	72.3 ± 4.2	76.1 ± 6.7	0.64
Sex (M:F)	2:29	0:2	0:4	1:3	1:21	
BMI (kg/m^2^) (mean ± SD)	25.74 ± 2.95	24.73 ± 4.28	22.33 ± 2.68	25.21 ± 2.22	25.74 ± 2.88	0.58
Limb-length discrepancy (mean ± SD)	12.76 ± 12.86	27.25 ± 14.41			7.24 ± 7.15	2.09

^a^Cohen’s d (0.2–0.5: small effect; 0.5–0.8: medium effect; > 0.8: large effect)

### Inclusion criteria for typical coxitis knee group

The parameters were adapted from previously published criteria and are as follows [9, 11, 12]:

1. Classic CK: Patients with an ankylosed hip due to either a pathological or surgical cause, with no prior knee pathology before hip fusion or degeneration. A hip adduction deformity with ipsilateral valgus knee.
2. LLA: Patients with CK exhibiting leg length discrepancy exceeding 2.5 cm, and a valgus knee deformity.
3. WSD: Patients with CK showing an accentuation of the valgus deformity on the affected side, and a varus knee degeneration on the contralateral side.

### Inclusion criteria for atypical coxitis knee group

Patients were categorized as atypical CK if they exhibited hip or knee pathologies that did not conform to the classic CK, LLA, or WSD diagnostic criteria.

The characterization of atypical CK is primarily based on the predominant hip condition, with a specific emphasis on identifying the presence of acetabular dysplasia, while also considering associated knee pathology.

### Exclusion criteria

Patients were excluded from the study if they met any of the following conditions:

1. Non-consenting patients: Patients who did not provide informed consent for their data to be used in the study.
2. Inadequate radiographic data: Patients with incomplete or insufficient quality radiographic data for assessing acetabular dysplasia and knee alignment.
3. Inadequate medical records: Patients with incomplete medical records lacking essential information for categorization and assessment.
4. Prior hip or knee arthroplasty: Patients with a history of prior hip or knee arthroplasty before the surgery under investigation.
5. Uncontrolled systemic conditions: Patients with uncontrolled systemic conditions or comorbidities that could significantly affect joint health or surgical outcomes.

### Statistical analysis

We used preoperative standard anteroposterior and lateral radiographs of the pelvis, hips, and knees, along with a full-length standing view of the lower limb, to assess various parameters and relevant radiographic changes. These parameters included the presence of acetabular dysplasia, defined as a center edge angle of less than 25 degrees, as well as knee alignment classified based on the Femoral-Tibial angle (FTA) into valgus (< 170), neutral (170–179), and varus (≥ 180). Additionally, we evaluated the degree of arthritis in both hips and knees using the Kellgren-Lawrence classification and identified the presence of subchondral insufficiency fractures. The threshold values for these parameters were adapted by our institution, considering local demographic information and research utilizing similar threshold values [17–19].

Categorical data were presented as frequencies and ratios to provide a comprehensive representation of patient groups and related variables. Continuous variables, including age, BMI, and leg-length discrepancy, were expressed as means with their respective standard deviation. We used Cohen’s d measure of effect size to compare the means of continuous variables, with 0.2 indicating a small effect, 0.5 a medium effect, and 0.8 or higher signifying a large effect. The association between acetabular dysplasia and the likelihood of presenting with an atypical CK was assessed using Fisher’s Exact Test. Additionally, we calculated the Odds Ratio (OR) with a 95% confidence interval (CI) to quantify this association. Computation was conducted using the Data Analysis ToolPak of Microsoft® Excel® for Microsoft 365 Version 2308.

## Results

Among the 31 individuals who had undergone both THA and TKA, only 10 patients (10:31) were categorized under typical CK, and were further broken down into classic (*n* = 2), LLAs (*n* = 4), and WSDs (*n* = 4). On the other hand, 21 patients (21:31) were classified under atypical CK. Comparing the leg length differences between the coxitis knee groups, we observed a significantly greater difference in leg length among typical CK patients (ranging from 12.9 to 41.6 mm). Conversely, the atypical CK patients showed a narrower range of leg length differences (from 0.1 to 14.4 mm). The effect size, as indicated by Cohen’s d, was notably larger for the typical CK group (Cohen’s d > 0.8), suggesting a substantial difference in leg length discrepancy within this cohort. In contrast, the effect size for the atypical CK group was smaller (Cohen’s d < 0.5), indicating a relatively smaller difference in leg length discrepancy among these patients (Table 1).

In the atypical CK group, there were 27 involved hips, among which acetabular dysplasia (*n* = 21), subchondral insufficiency fracture-like changes (*n* = 5), and non-dysplastic classic hip arthritic changes (*n* = 1) were identified (Table 2). Of the 27 knees that underwent surgery, 26 exhibited varus alignment, and 1 had normal alignment. None of the knees in this group showed valgus alignment. Among the 21 patients, TKAs were performed on the ipsilateral (*n* = 1), contralateral (*n* = 14), and bilateral (*n* = 6) knees. For the non-operated side (*n* = 15), 4 knees had almost normal radiographs with minimal to no symptoms, while 11 knees showed tolerable symptoms and medial compartment arthritis (Table 3).

**Table 2:** Hip breakdown in the atypical CK group of 21 patients

**(21 Patients)**		**Frequency**
Involved hip pathology (*n* = 27 hips)	Acetabular dysplasia	*n* = 21:27
	Subchondral insufficiency fracture-like change	*n* = 5:27
	Classic degenerative hip arthritis	*n* = 1:27

**Table 3:** Knee breakdown in the atypical CK group of 21 patients

**(21 Patients)**		**Laterality**		
	(Patients, [knees])	Ipsilateral (1:21)	Contralateral (14:21)	Bilateral (6:21)
Alignment (*n* = 27 operated knees)	Varus (*n* = 26:27)	1, [1]	13, [13]	6, [12]
	Normal (*n* = 1:27)	0	1 [1]	0
	Valgus (*n* = 0:27)	0	0	0
Contralateral knee status (*n* = 15 knees)		(Knees)		
	None-mild symptoms	4:15		
	Early arthritis (KL 1–2)	5:15		
	Late arthritis (KL 3–4)	6:15		

*KL* Kellgren-Lawrence grading

The Fisher’s Exact Test revealed a significant association between acetabular dysplasia and atypical CK (*P* = 0.00997). Patients with acetabular dysplasia had 8.2 times higher odds of presenting with atypical CK (OR: 8.2, 95% CI: 1.6–41.6) (Table 4).

**Table 4:** Odds associated with acetabular dysplasia to atypical coxitis knee among 31 patients

	**Atypical CK**	**Typical CK**
Acetabular dysplasia	21	3
Non-dysplastic hips	6	7
Fisher’s exact probability	0.00997	
Level of significance	5% (0.05)	
Odds Ratio (OR)	8.2 (95% CI [1.6, 41.6])	

## Discussion

Our study focused on a distinct subset of CK patients. Specifically, individuals with pre-existing acetabular dysplasia and varus knee configuration present a unique variant, which we term “Acetabular Dysplasia Associated Gonarthritis (ADAG)”. In this variant, disease progression unfolds as follows: Initially, hip pain prompts a reduction in the load on the affected knee by at least 18% of the peak ground reaction force [20]. This load reduction, aimed at unloading the arthritic hip, triggers chronic weight-bearing asymmetry, ultimately leading to osteoarthritis in the contralateral knee [21].

**Fig. 1:**
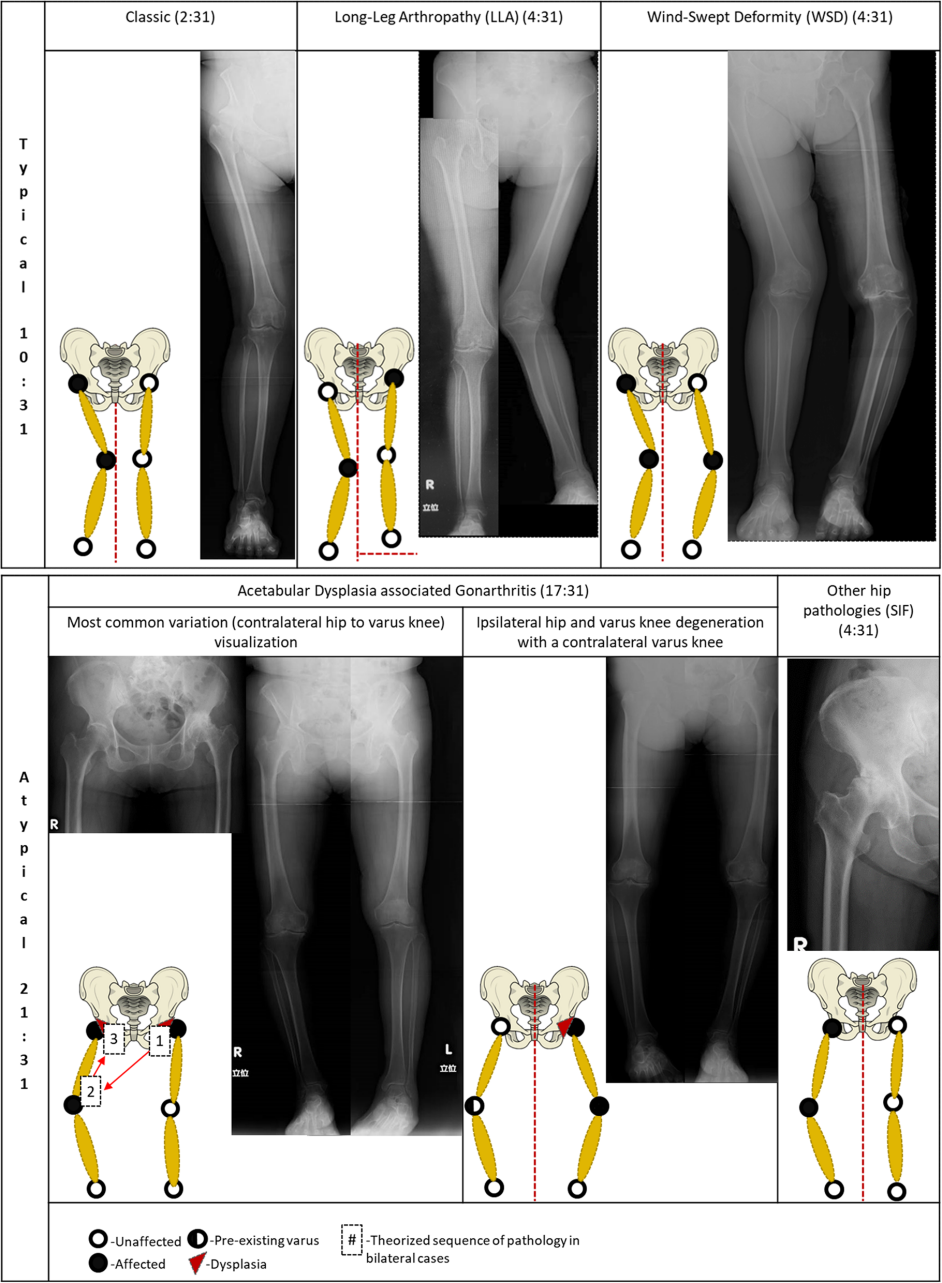
Summary diagram and representatives Coxitis Knee classification among 31 patients

For patients with AD, the superior-inferior and medial–lateral hip movements significantly affect the knee adduction moment on the contralateral side [21, 22]. The increased moment, stemming from a more lateral hip center, places substantial load on the medial compartment of the contralateral knee, resulting in further degeneration and a varus knee position [22]. As the knee condition deteriorates, external rotation tendencies become evident [23]. This tendency complements the ascending kinematic chain, predisposing the knee to extension and external rotation [24]. These motions translate proximally, leading to extension, abduction, and external rotation of the ipsilateral hip, causing posterior pelvic tilting [24]. Retroversion decreases femoral head coverage, disturbing stress distribution [25]. Patients with AD compensate for this deficiency by tilting the pelvis anteriorly, setting in motion a contradictory action between the movement chain of the dysplastic hip and varus knees, accelerating hip and knee osteoarthritis progression on both sides [25–27] (Fig. 1).

Hauge et al., Dixon et al., and Smyth et al. elucidated the mechanism underlying typical coxitis knee (CK), emphasizing the adduction deformity of the hip, the axis deviation of the valgus knee, overloading in a relatively lengthened limb, and the effect on the contralateral knee [9, 11, 12]. However, the contribution of acetabular dysplasia to knee degeneration received limited attention in the literature, leaving their pathophysiological underpinnings less explored. Gullo et al. acknowledged the relationship between hip and knee osteoarthritis in classic CK, yet the mechanisms through which acetabular dysplasia influences knee degeneration remained unclear [2]. Notably, Matsuyama et al. and Murata et al. mainly focused on spine associations with hip or knee arthritis, given the higher standardized frequency of spine degeneration compared to hip and knee osteoarthritis [2–4, 6]. Another factor is that the previous associations were lumped together with multiple osteoarthritis, mainly because the diagnosis is not clear or established [2, 6]. Shinsuke et al. studied some associations of hip and knee arthritis, similar to the original proponents, concentrating mainly on classifying into leg-length arthropathy and wind-swept deformity [13, 18]. Although their focus was on patients with unilaterally dislocated hips, they provided valuable insights by highlighting that patients with acetabular dysplasia have an increased predilection to load the medial side of the contralateral knee [13, 14, 21].

The strength of this study lies in its exploration of “acetabular dysplasia-associated gonarthritis” as a unique and previously understudied variant of coxitis knee. By investigating the intricate interplay among factors such as lateralized hip center, chronic weight-bearing asymmetry, knee-adduction moment changes, and the subsequent development of varus knee degeneration within the context of kinematic chains, our research advances our understanding of CK pathophysiology [21, 22]. Furthermore, our study contributes by expanding upon and confirming established knowledge related to classic CK. Our findings underscore the clinical relevance of recognizing CK subtypes, as it may lead to more tailored diagnostic and therapeutic approaches, potentially improving patient care.

The study’s relatively small cohort size, driven by strict inclusion criteria reflecting the condition’s rarity, presented practical constraints in patient recruitment despite our efforts to identify eligible cases. Due to a retrospective observational cohort design, the study may encounter inconsistencies in data collection, although we attempted to mitigate this through systematic documentation. Our primary focus on patients with acetabular dysplasia led to a comprehensive exploration of this majority group, while limiting an in-depth investigation of other subsets like subchondral insufficiency fractures, fragility, and osteonecrosis to maintain overall coherence. Furthermore, the study’s exclusive reliance on data from a single hospital, which specializes in treating such patients, may potentially limit its generalizability as the results might not fully encompass the demographic diversity observed in larger multi-center studies. Given the complexities and variations observed in patients with hip and knee osteoarthritis, the authors recommend conducting a multi-center study once the diagnosis is established. Such a study would involve a larger and more diverse population, which could provide a more comprehensive understanding of the prevalence and course of this variant. This approach may help validate the findings of this study and contribute to a more robust understanding of the relationship between hip dysplasia, varus knee degeneration, and the associated clinical outcomes.

## Conclusion

The prevalence of acetabular dysplasia-associated gonarthritis as a distinctive variant of CK is relevant in populations with high acetabular dysplasia rates. Identifying CK subtypes is crucial for tailored treatment and improved clinical outcomes.

## Data Availability

The data that support the findings of this study are available from the corresponding author upon reasonable request, but restrictions apply to the availability of these data, which were used under regulation by national law, and so are not publicly available.

## Abbreviations

CK: Coxitis Knee
THA: Total Hip Arthroplasty
TKA: Total Knee Arthroplasty
LLA: Long-Leg Arthropathy
WSD: Wind-Swept Deformity
FTA: Femoral Tibial Angle
ADAG: Acetabular Dysplasia Associated Gonarthritis
SIF: Subchondral Insufficiency Fracture
